# Supplementary material for: The effect of single mutations in Zika virus envelope on escape from broadly neutralizing antibodies
Source: J Virol. 2023 Nov 9;97(11):e01414-23. doi: 10.1128/jvi.01414-23 (PMC10688354; doi:10.1128/jvi.01414-23)
Supplement: Supplemental figures and tables — Figures S1 to S11, Tables S1 to S6. [file jvi.01414-23-s0001.docx]

# Supplementary Information

## Supplemental figure 1. Correlations across replicate deep mutational scanning experiments

##
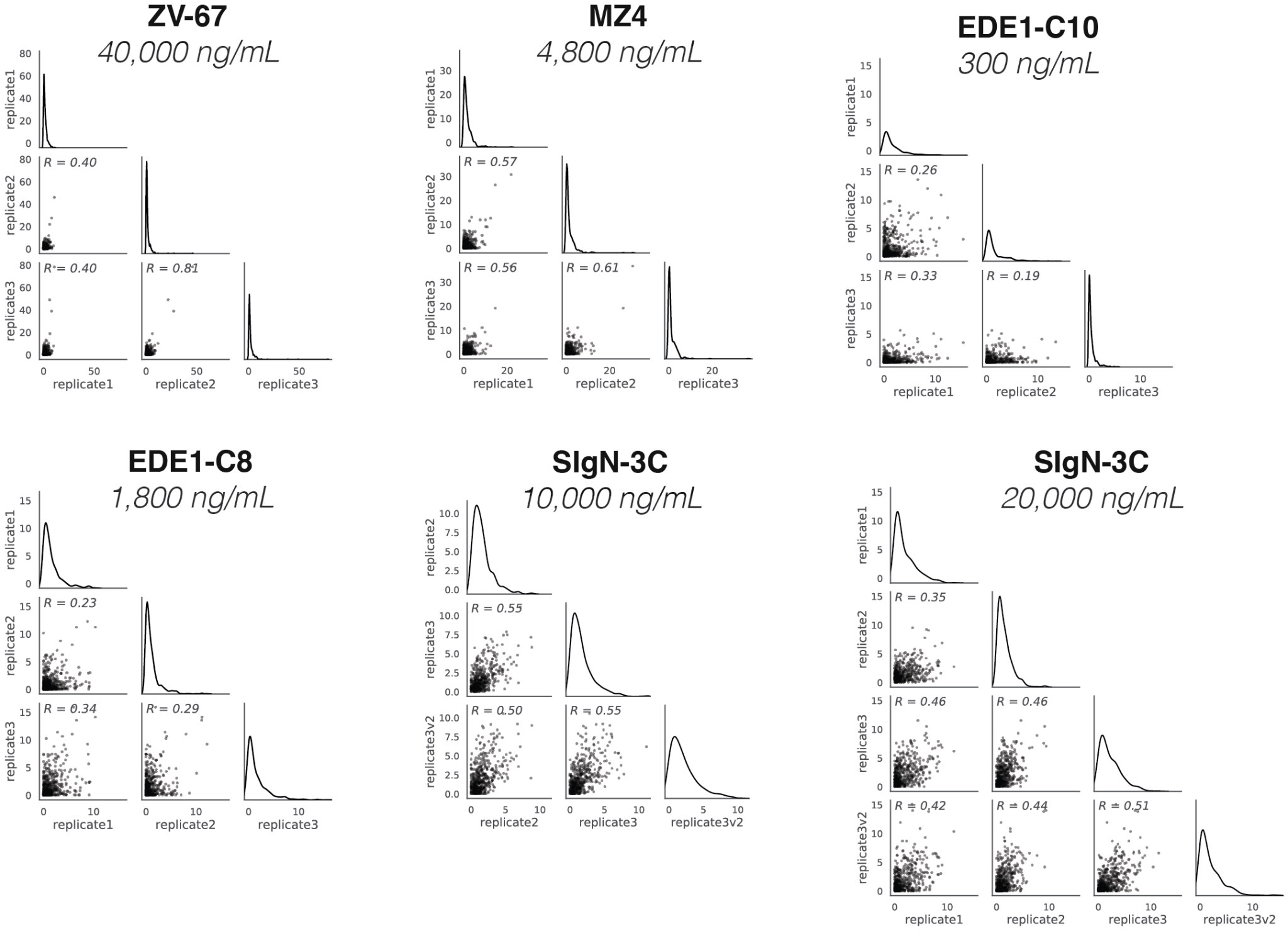


Points represent site-wise differential selection (our metric of antibody escape) across all amino-acid mutations. The replicates correlate well for narrow antibodies, where a few sites lead to high degrees of escape. For broad antibodies, where no sites lead to large magnitudes of escape, the results are reasonably correlated.

## Supplemental figure 2. Mutation-level deep mutational scanning from ZV-67 selections across the entire E protein.

*
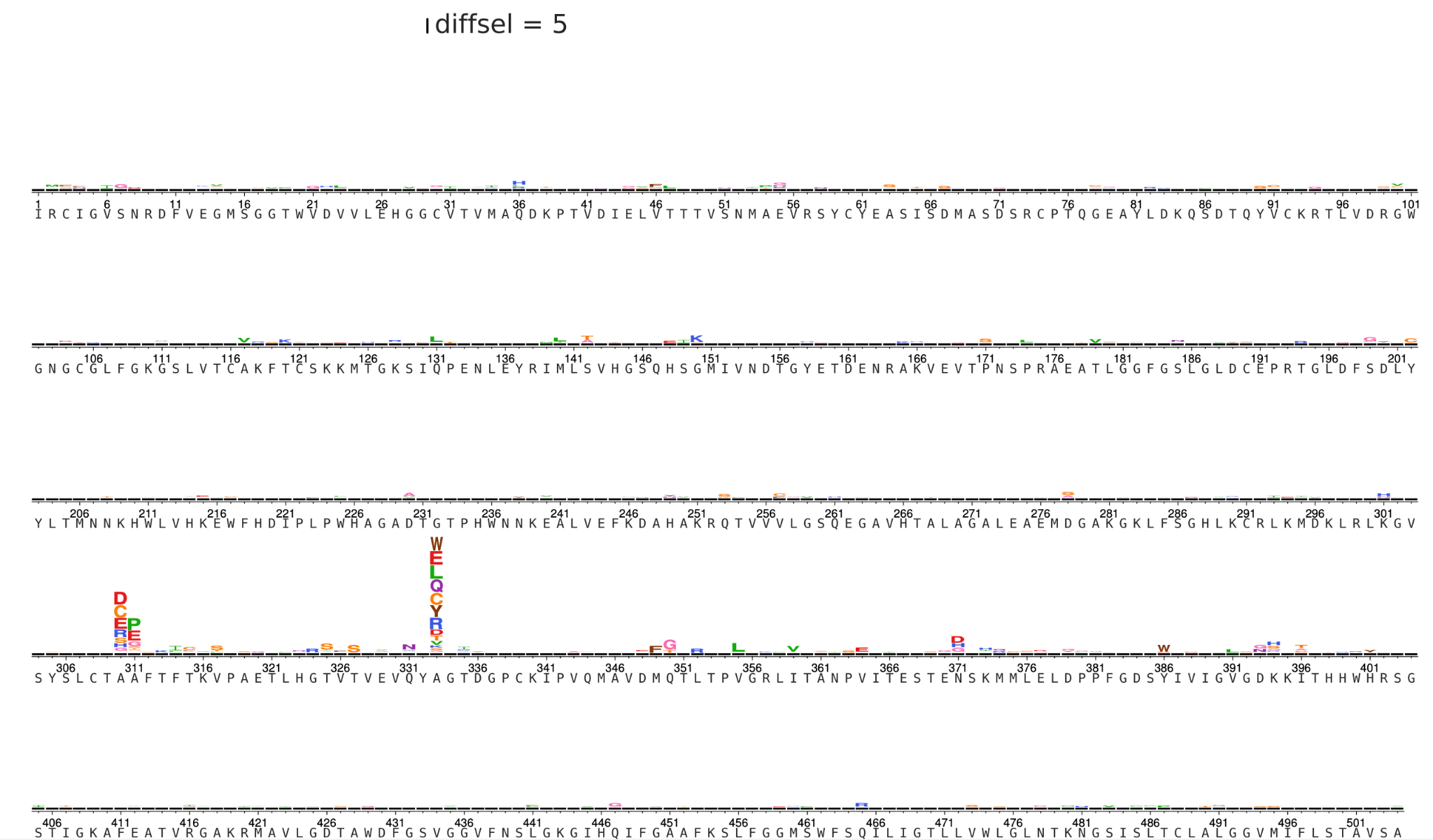
*

The effect of single amino acid mutations on neutralization, shown as logo plots where the height of the letter is scaled to the magnitude of antibody escape attributed to that amino acid mutation. The plot is scaled by differential selection (diffsel), our metric of antibody escape. See **Methods** for more details.

## Supplemental figure 3. Mutation-level deep mutational scanning from MZ4 selections across the entire E protein.

**
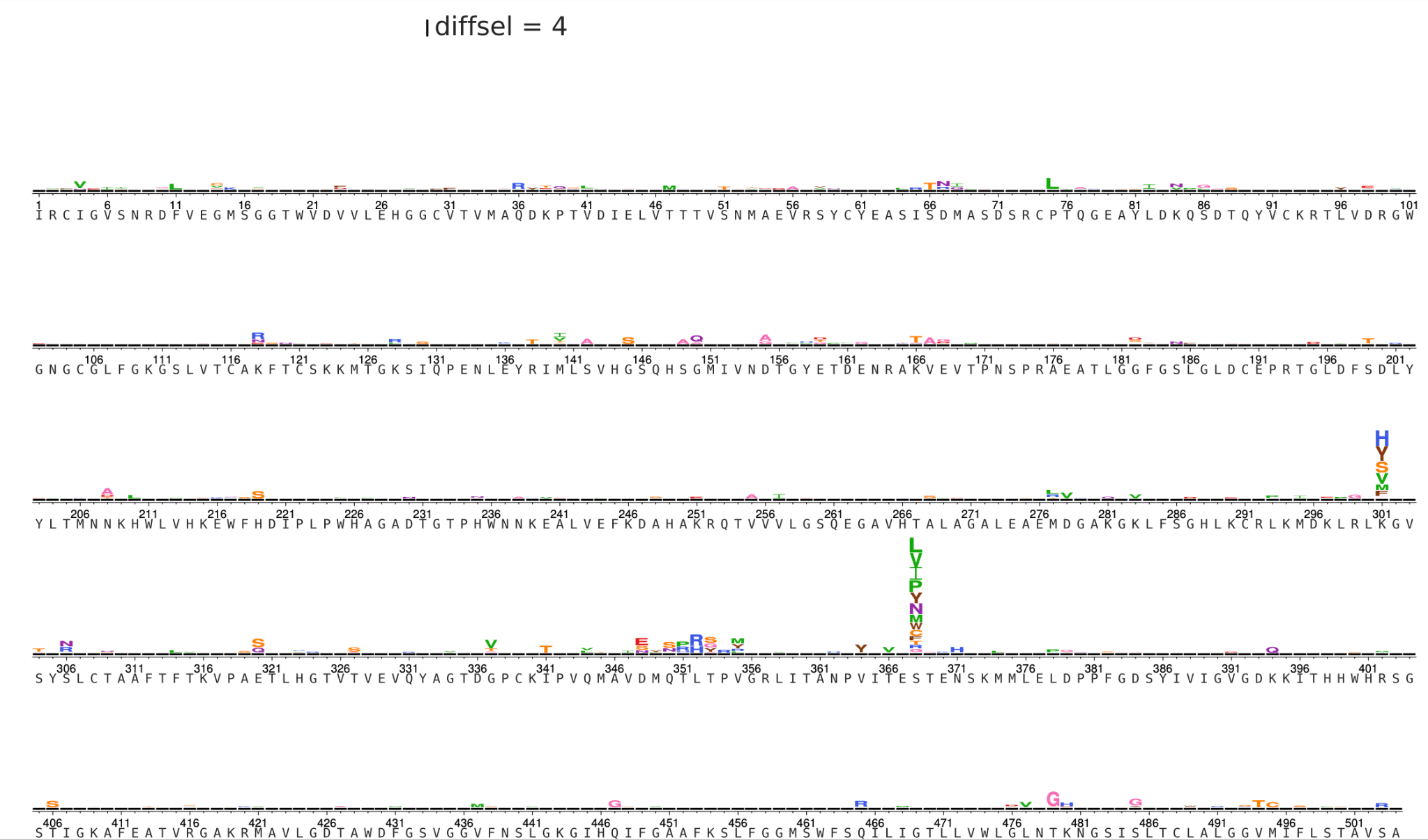
**

## The effect of single amino acid mutations on neutralization, shown as logo plots where the height of the letter is scaled to the magnitude of antibody escape attributed to that amino acid mutation. The plot is scaled by differential selection (diffsel), our metric of antibody escape. See Methods for more details.

## Supplemental figure 4. Mutation-level deep mutational scanning from EDE1-C10 selections across the entire E protein.

*
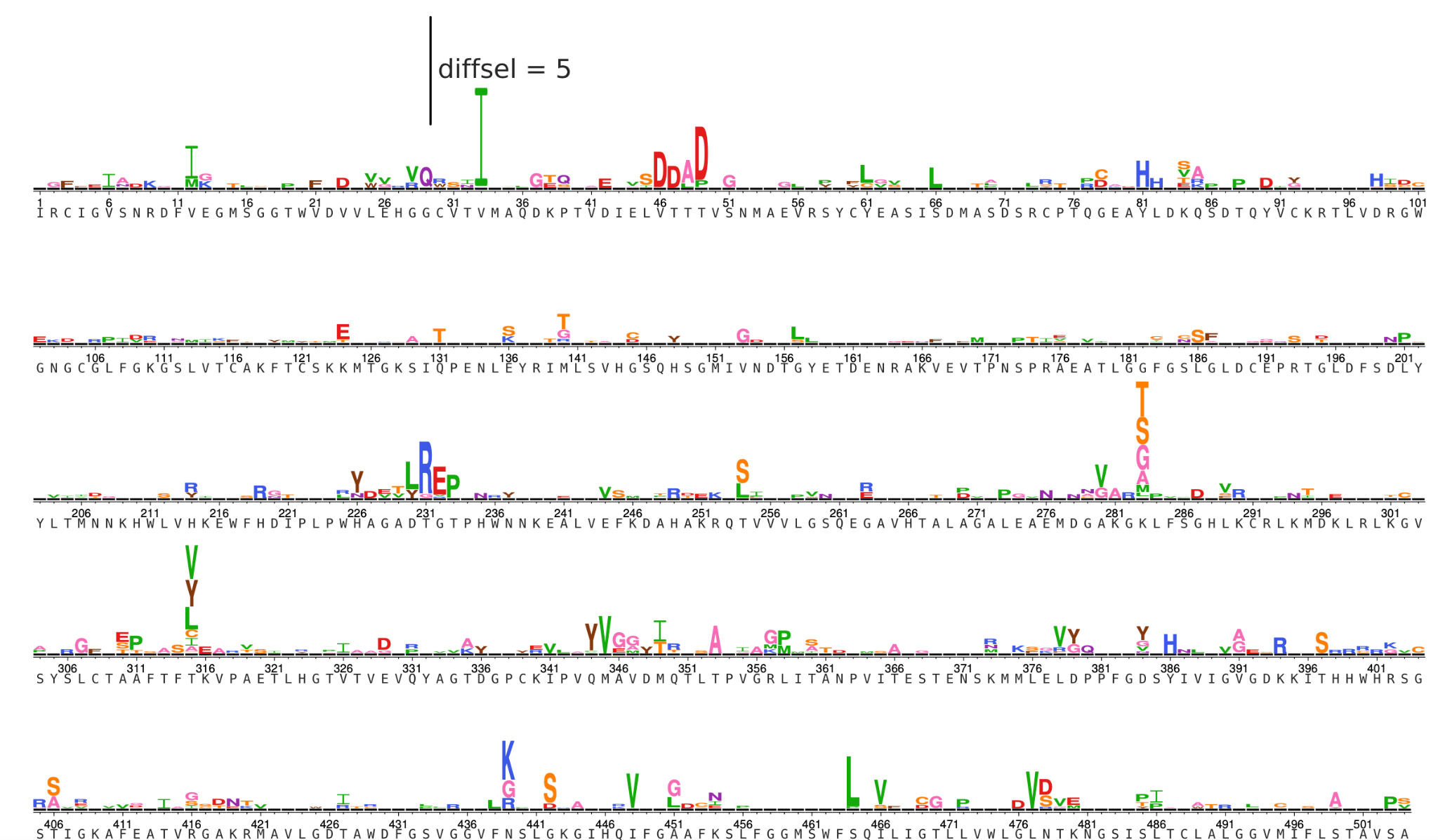
*

The effect of single amino acid mutations on neutralization, shown as logo plots where the height of the letter is scaled to the magnitude of antibody escape attributed to that amino acid mutation. The plot is scaled by differential selection (diffsel), our metric of antibody escape. See **Methods** for more details.

## Supplemental figure 5. Mutation-level deep mutational scanning from EDE1-C8 selections across the entire E protein.

*
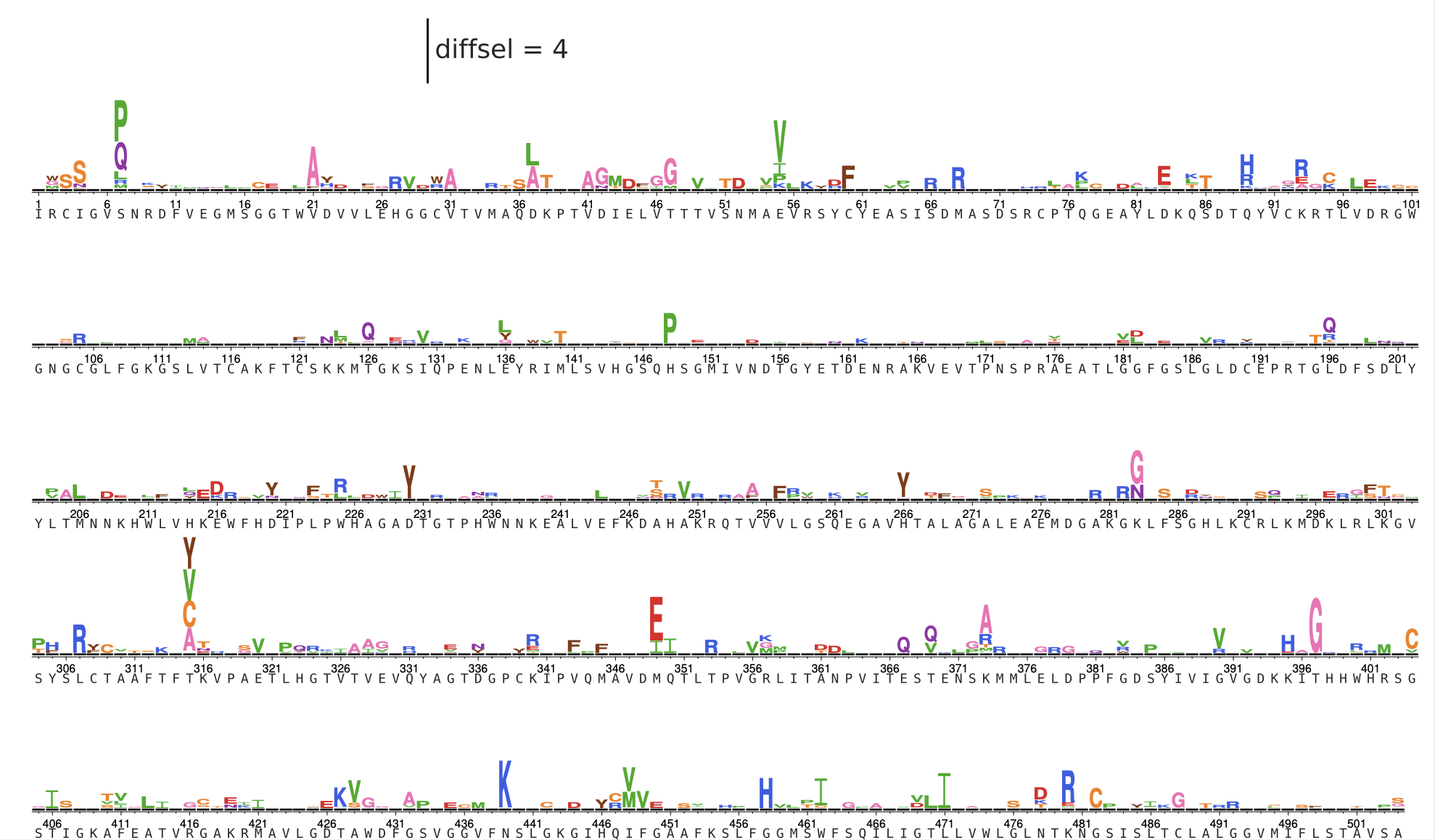
*

The effect of single amino acid mutations on neutralization, shown as logo plots where the height of the letter is scaled to the magnitude of antibody escape attributed to that amino acid mutation. The plot is scaled by differential selection (diffsel), our metric of antibody escape. See **Methods** for more details.

## Supplemental figure 6. Mutation-level deep mutational scanning from SIgN-3C selections across the entire E protein.

*
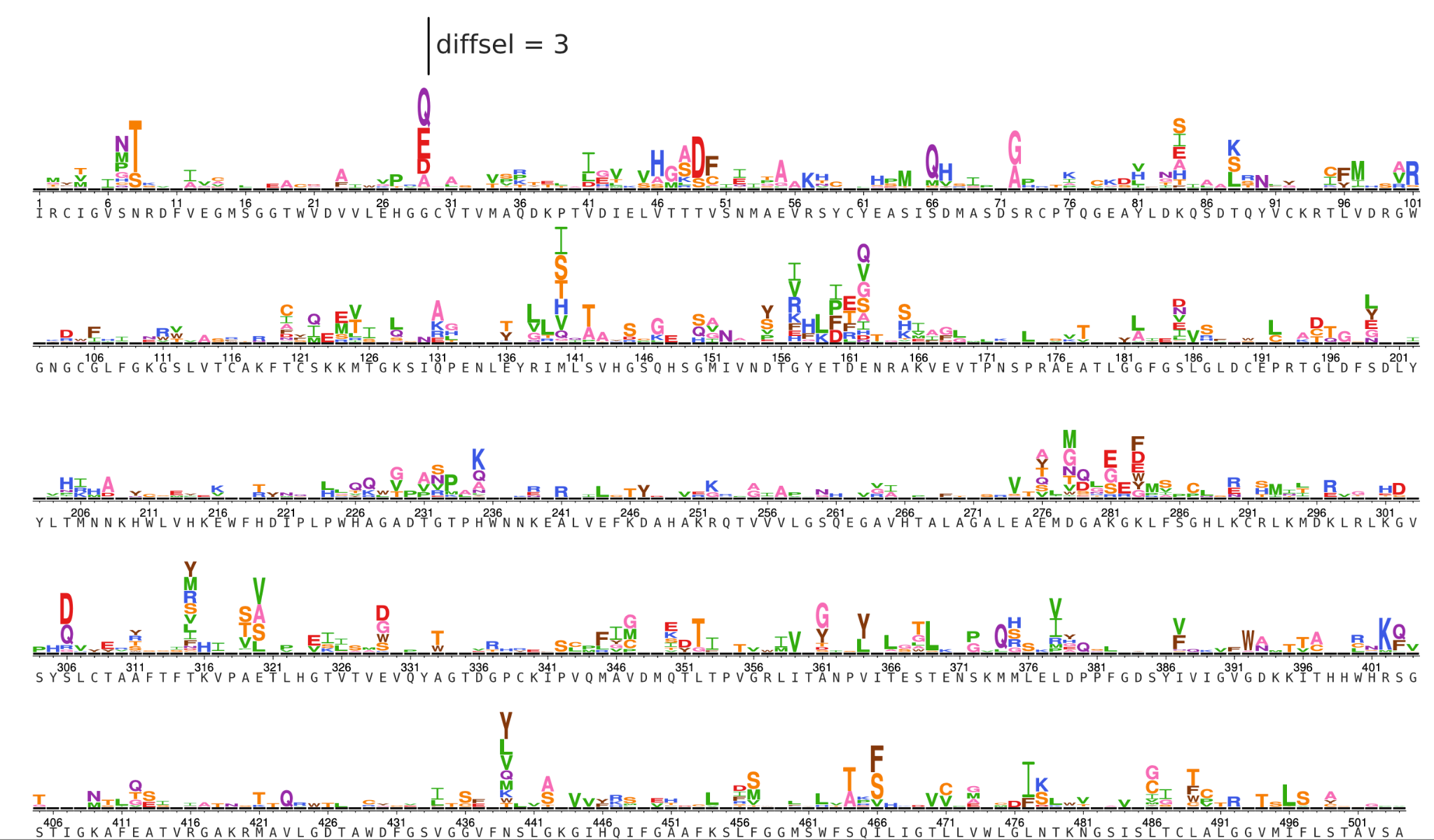
*

The effect of single amino acid mutations on neutralization, shown as logo plots where the height of the letter is scaled to the magnitude of antibody escape attributed to that amino acid mutation. The plot is scaled by differential selection (diffsel), our metric of antibody escape. See **Methods** for more details.

##

## Supplemental figure 7. Flavivirus E protein alignment

##
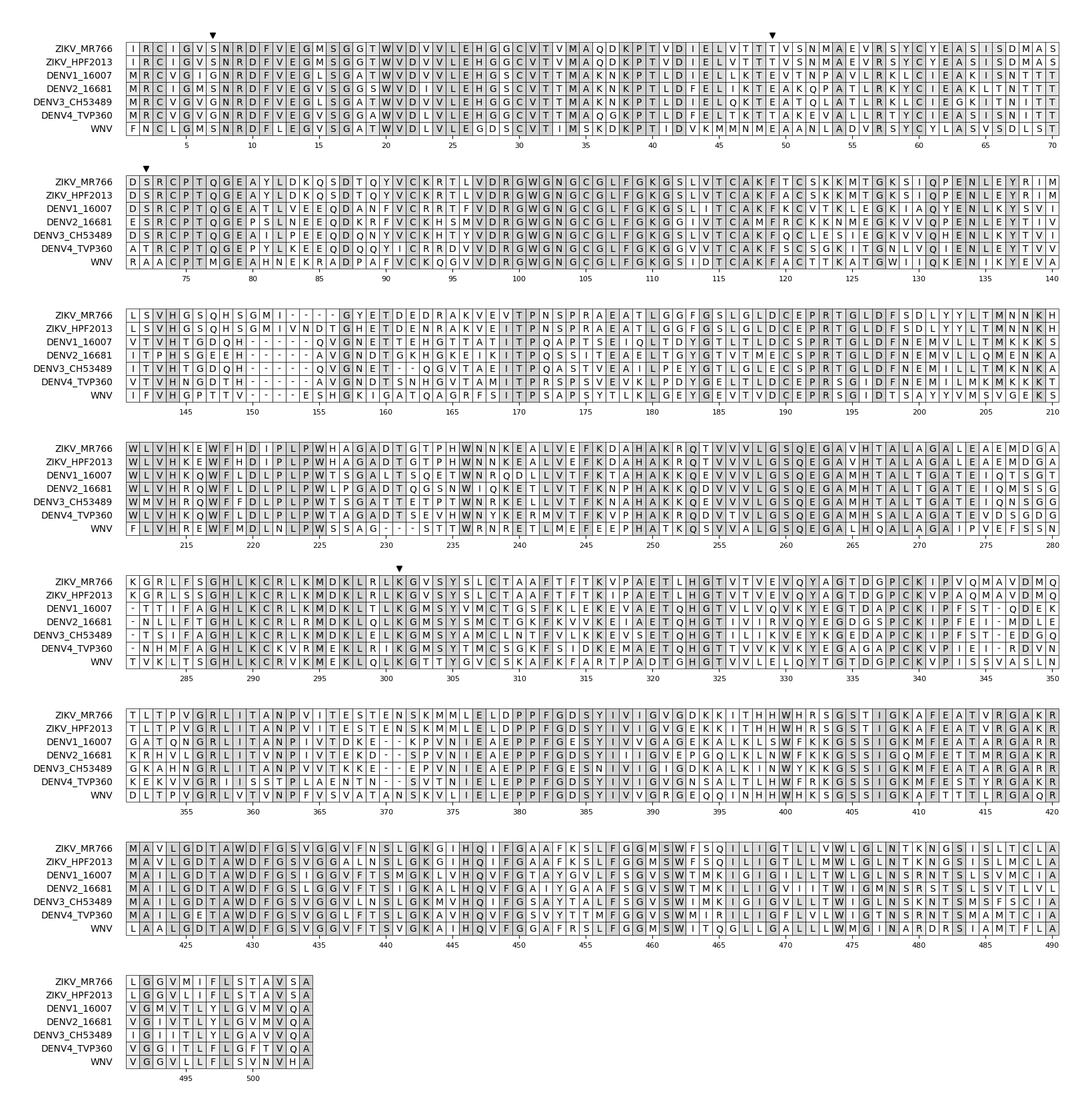


Black arrows indicate sites where site-directed mutagenesis was targeted, and light gray highlight indicates site percent identity. See **Methods** for more details on sequences used and how the alignment was generated.

## Supplemental figure 8. Individual Hill Curves interpolated for technical triplicate neutralization assays with Zika virus MR766

*
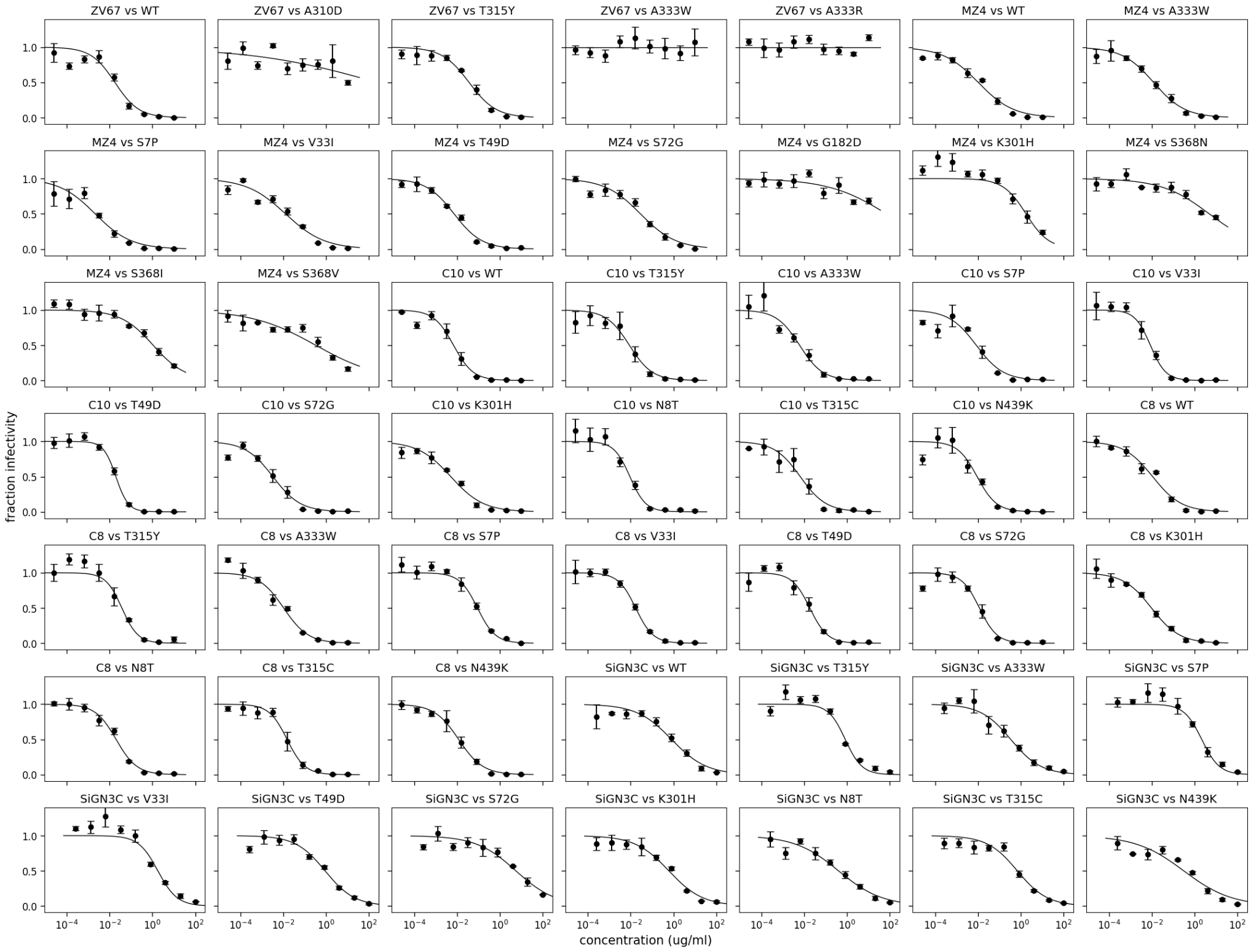
*

Points indicate the mean and standard error across three technical replicates.

## Supplemental figure 9. Site-level escape for all antibodies plotted against site-level neffective


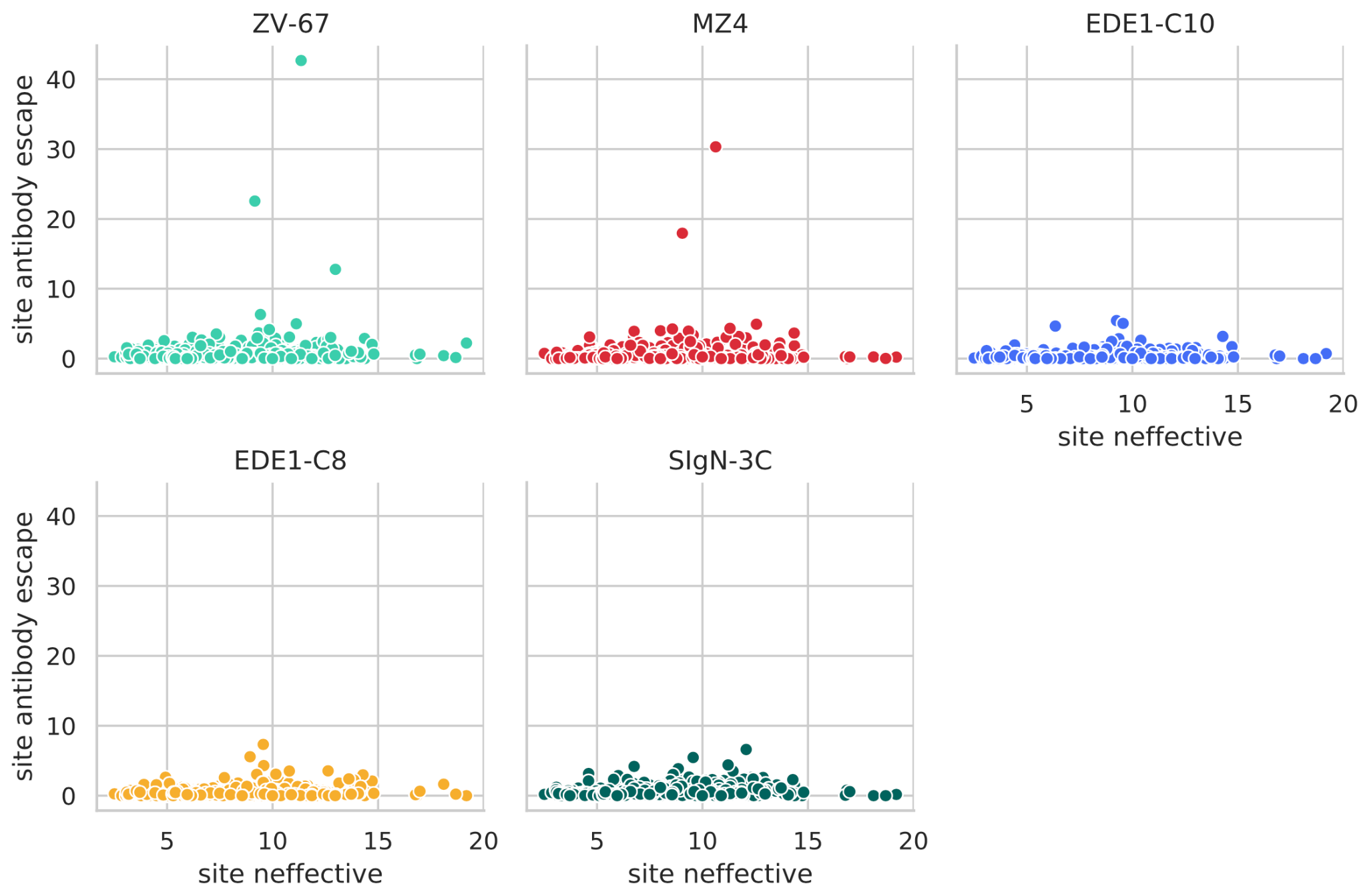


Site-wise summed antibody escape is plotted against a metric of site mutational tolerance, neffective. See **Methods** for more details.

## Supplemental figure 10. Individual Hill Curves interpolated for technical triplicate neutralization assays with Zika virus H/PF/2013


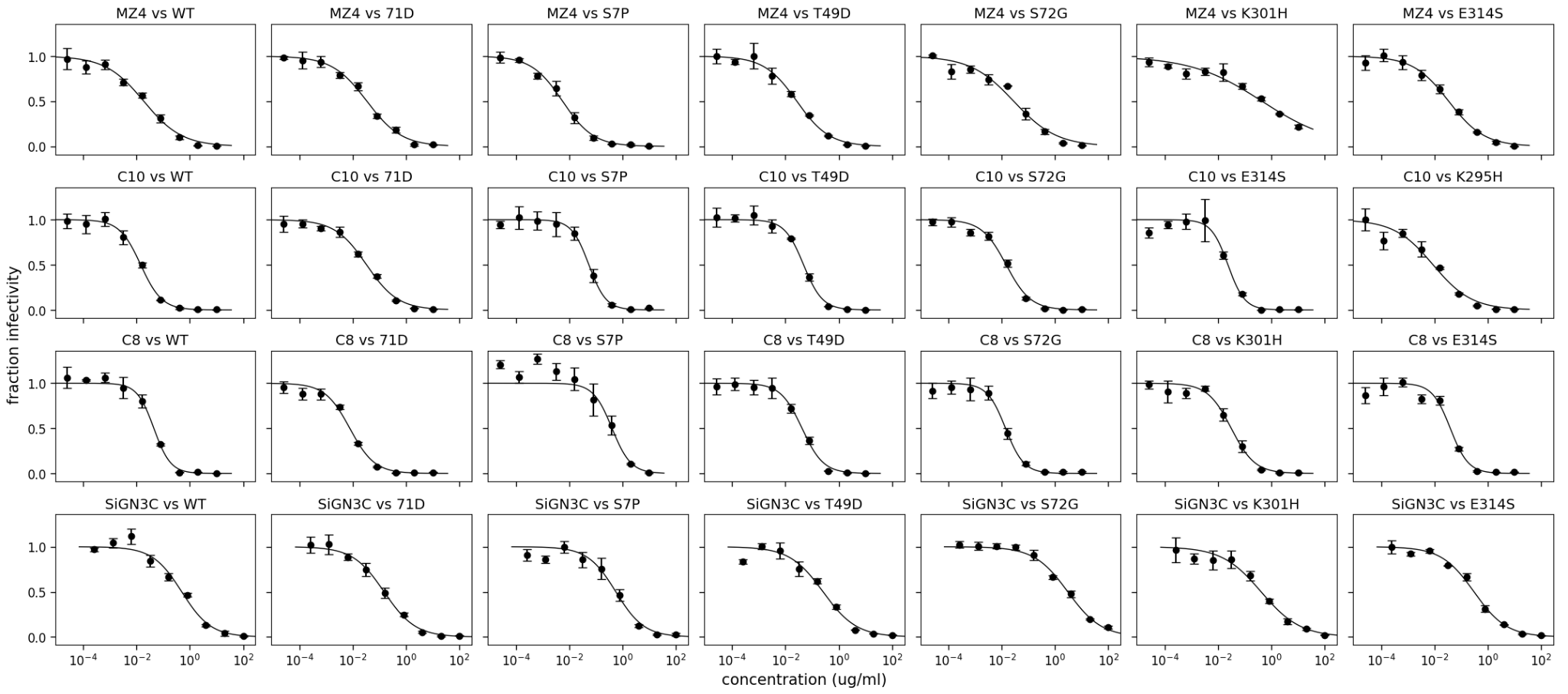


Points indicate the mean and standard error across three technical replicates.

## Supplemental figure 11. Individual Hill Curves interpolated for technical triplicate neutralization assays with dengue virus serotype 2 16681

##
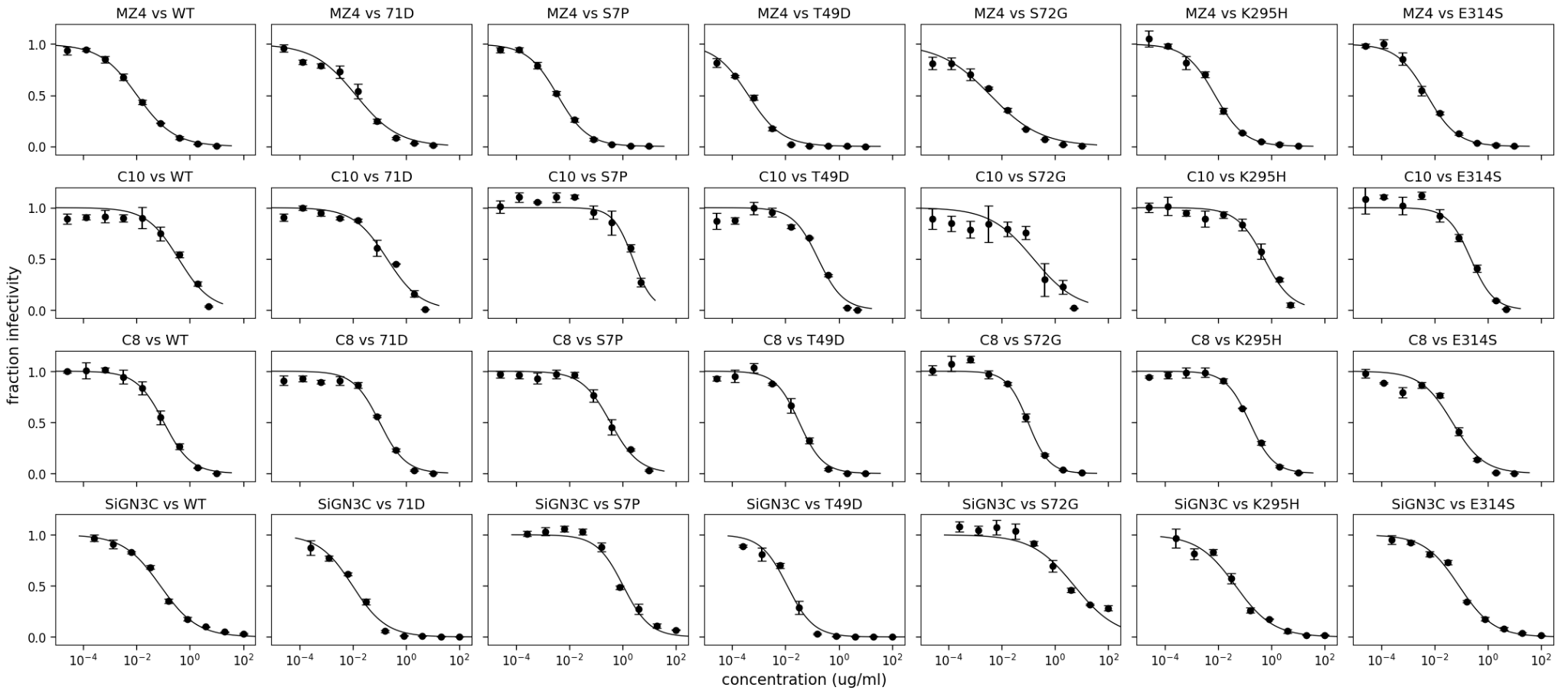


Points indicate the mean and standard error across three technical replicates.

##

## Supplemental table 1. Antibody concentrations used to neutralize MR766 Zika virus DMS libraries


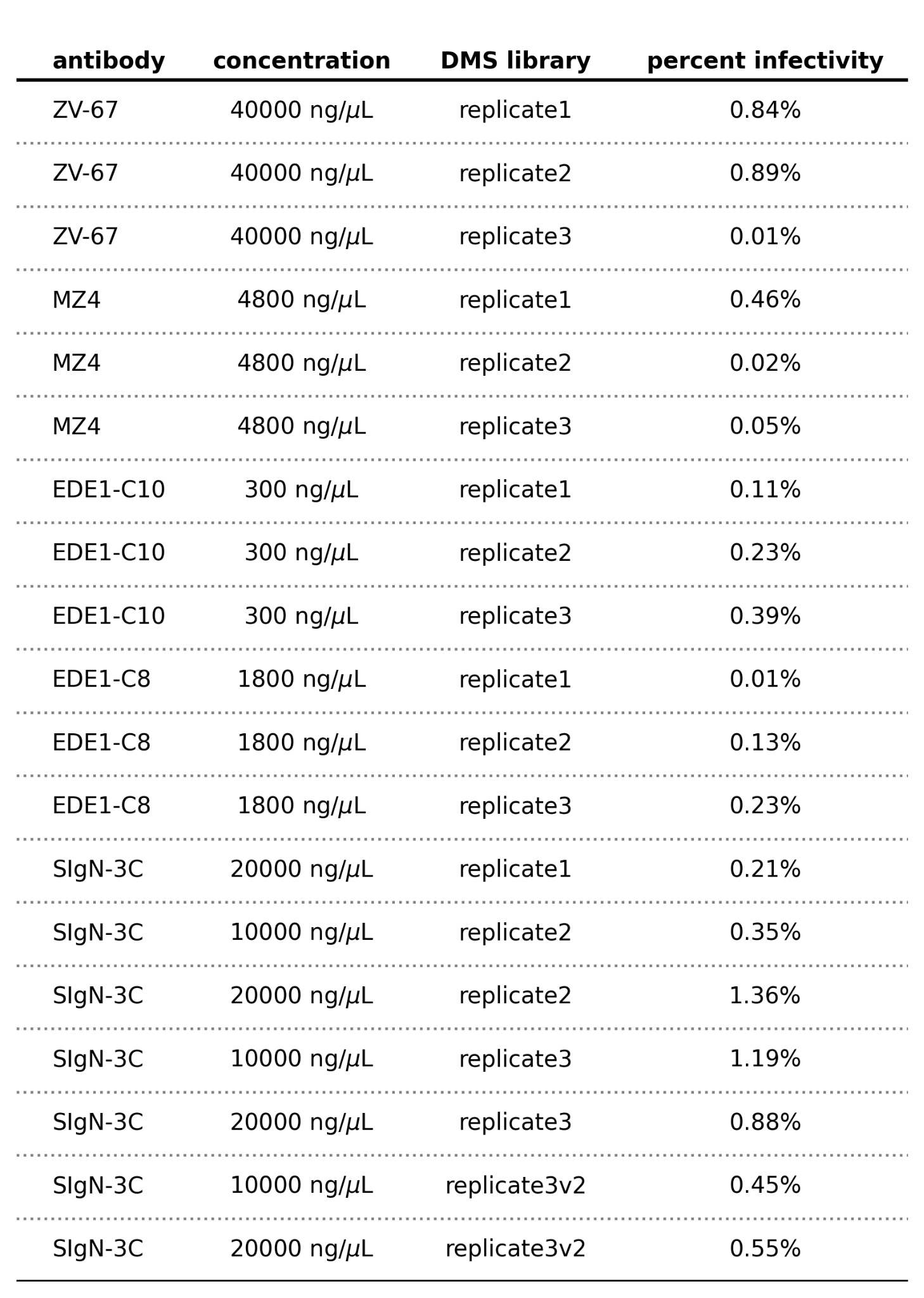


Antibodies were incubated at the indicated concentration with the indicated biological replicate deep mutational scanning (DMS) library. The percent infectivity was quantified by qRT-PCR. See **Methods** for details.

## Supplemental table 2. IC50s and statistical tests for antibody ZV-67


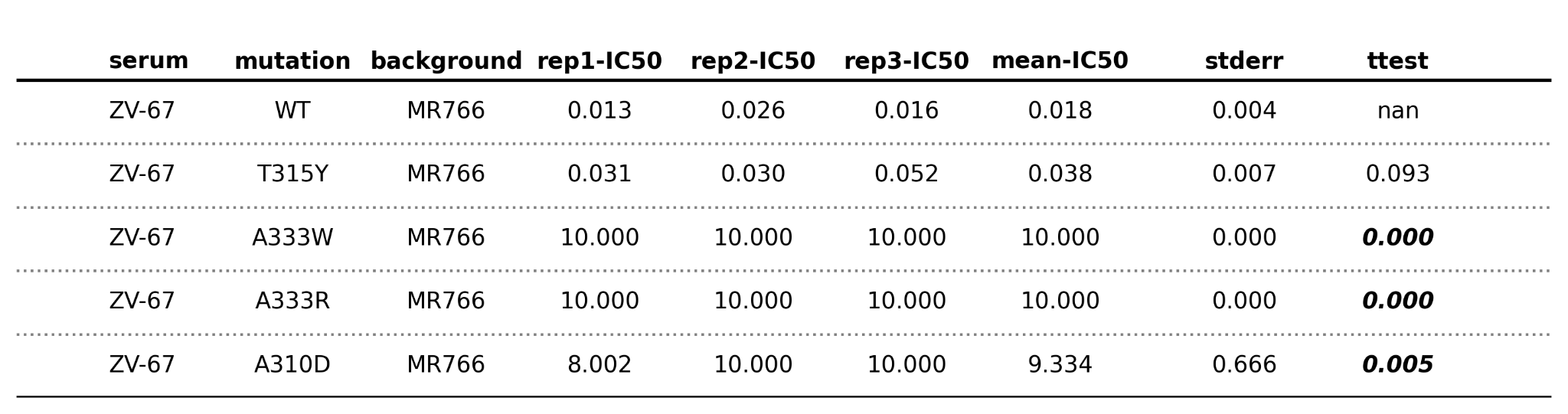


Antibodies were incubated with viruses containing the indicated mutation created in the indicated viral background. Hill curves and IC50s were inferred in technical triplicate, and the mean and standard error (stderr) were calculated. To assess for differences between wild-type (WT) and mutant virus IC50s, a Student’s T test was performed (ttest). See **Methods** for details.

## Supplemental table 3. IC50s and statistical tests for antibody MZ4


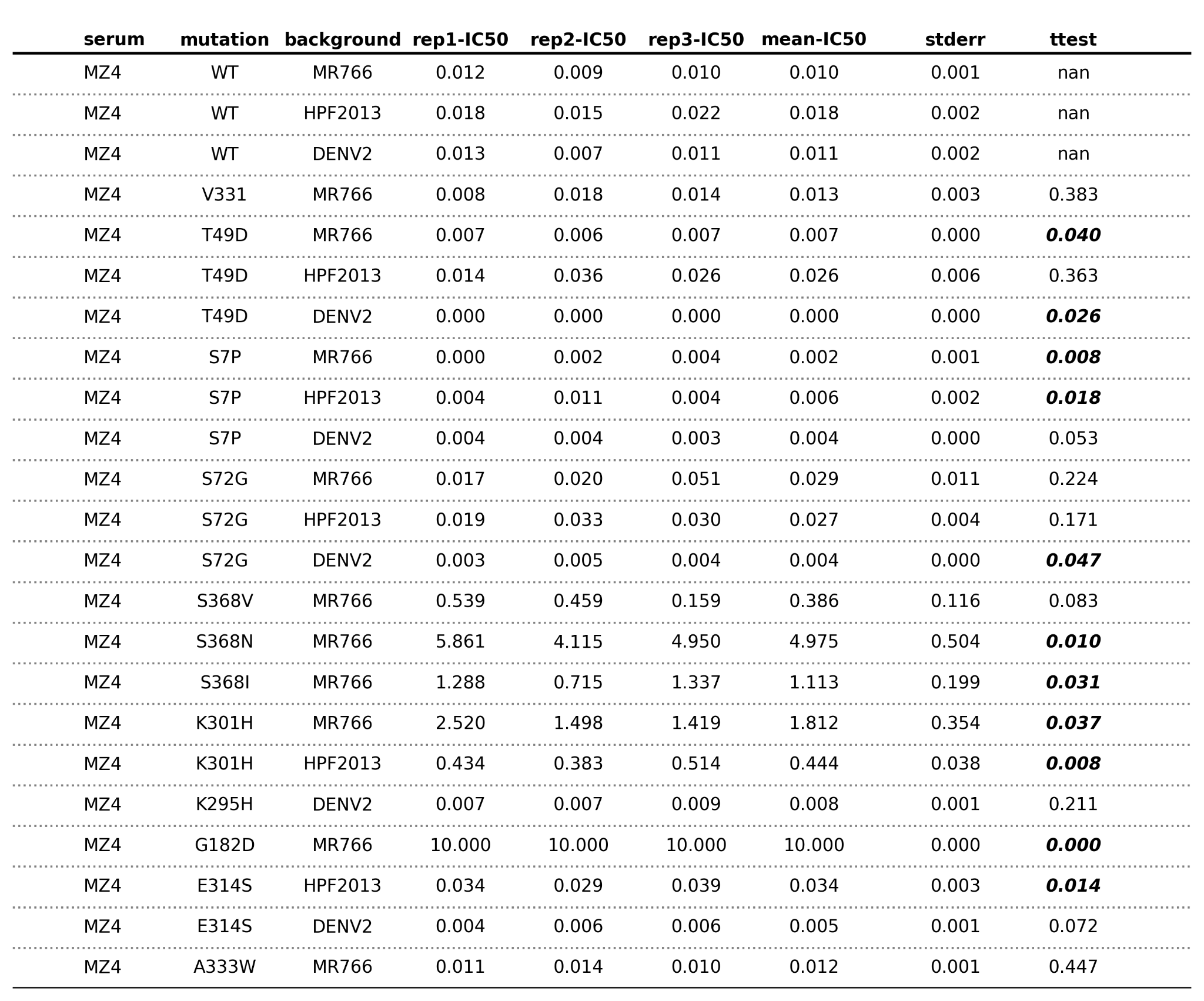


Antibodies were incubated with viruses containing the indicated mutation created in the indicated viral background. Hill curves and IC50s were inferred in technical triplicate, and the mean and standard error (stderr) were calculated. To assess for differences between wild-type (WT) and mutant virus IC50s, a Student’s T test was performed (ttest). See **Methods** for details.

## Supplemental table 4. IC50s and statistical tests for antibody EDE1-C10


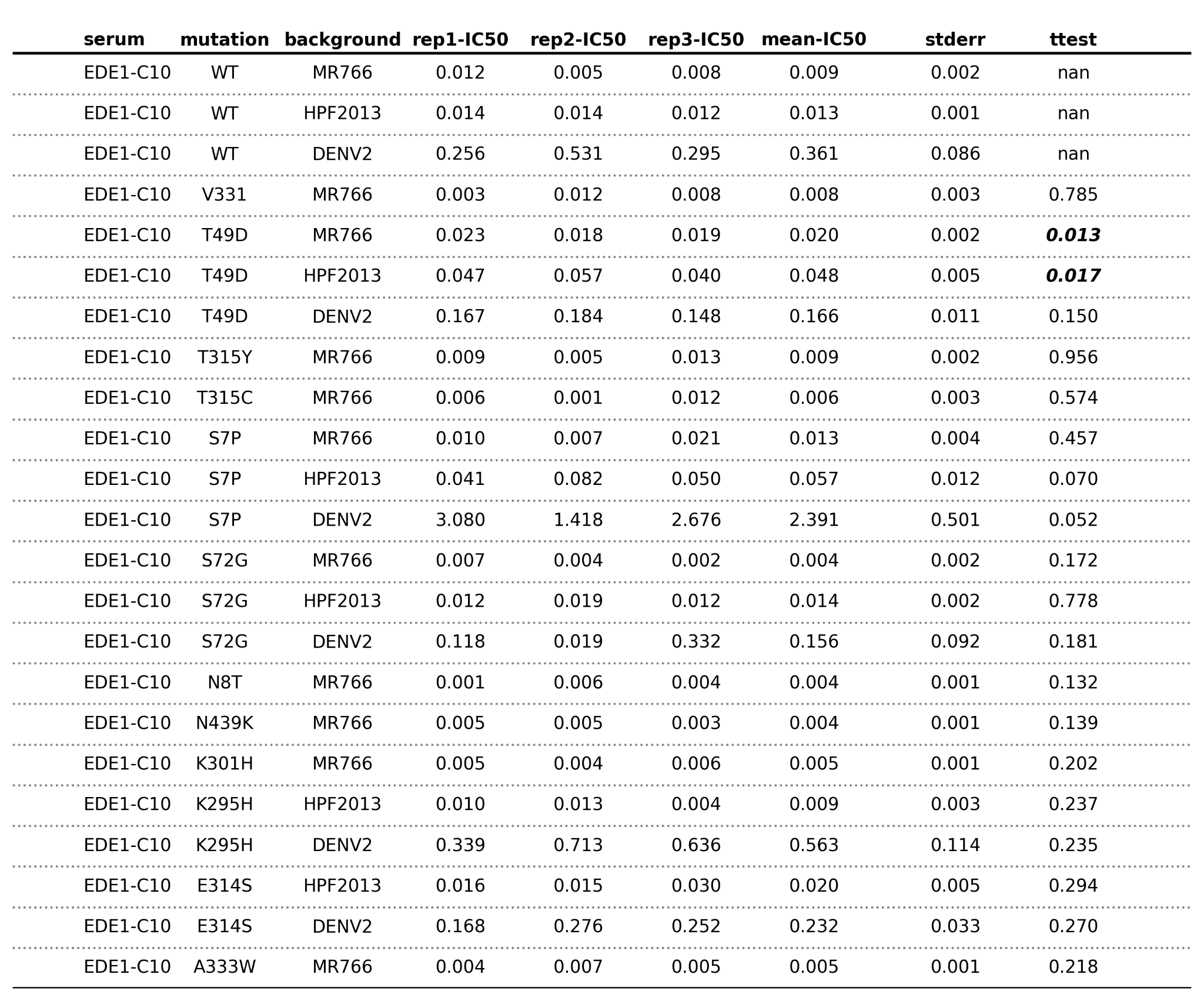


Antibodies were incubated with viruses containing the indicated mutation created in the indicated viral background. Hill curves and IC50s were inferred in technical triplicate, and the mean and standard error (stderr) were calculated. To assess for differences between wild-type (WT) and mutant virus IC50s, a Student’s T test was performed (ttest). See **Methods** for details.

## Supplemental table 5. IC50s and statistical tests for antibody EDE1-C8


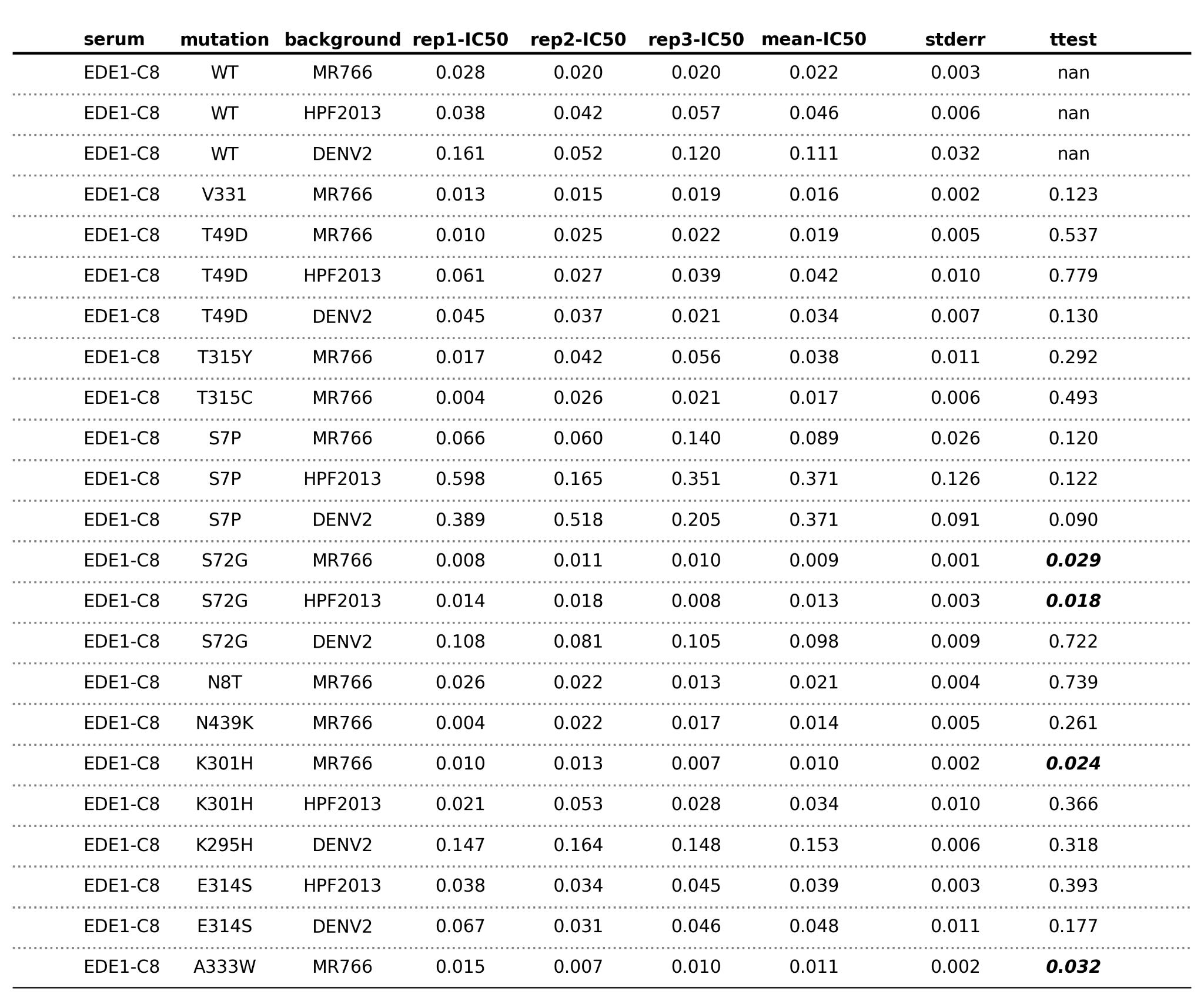


Antibodies were incubated with viruses containing the indicated mutation created in the indicated viral background. Hill curves and IC50s were inferred in technical triplicate, and the mean and standard error (stderr) were calculated. To assess for differences between wild-type (WT) and mutant virus IC50s, a Student’s T test was performed (ttest). See **Methods** for details.

## Supplemental table 6. IC50s and statistical tests for antibody SIgN-3C

#
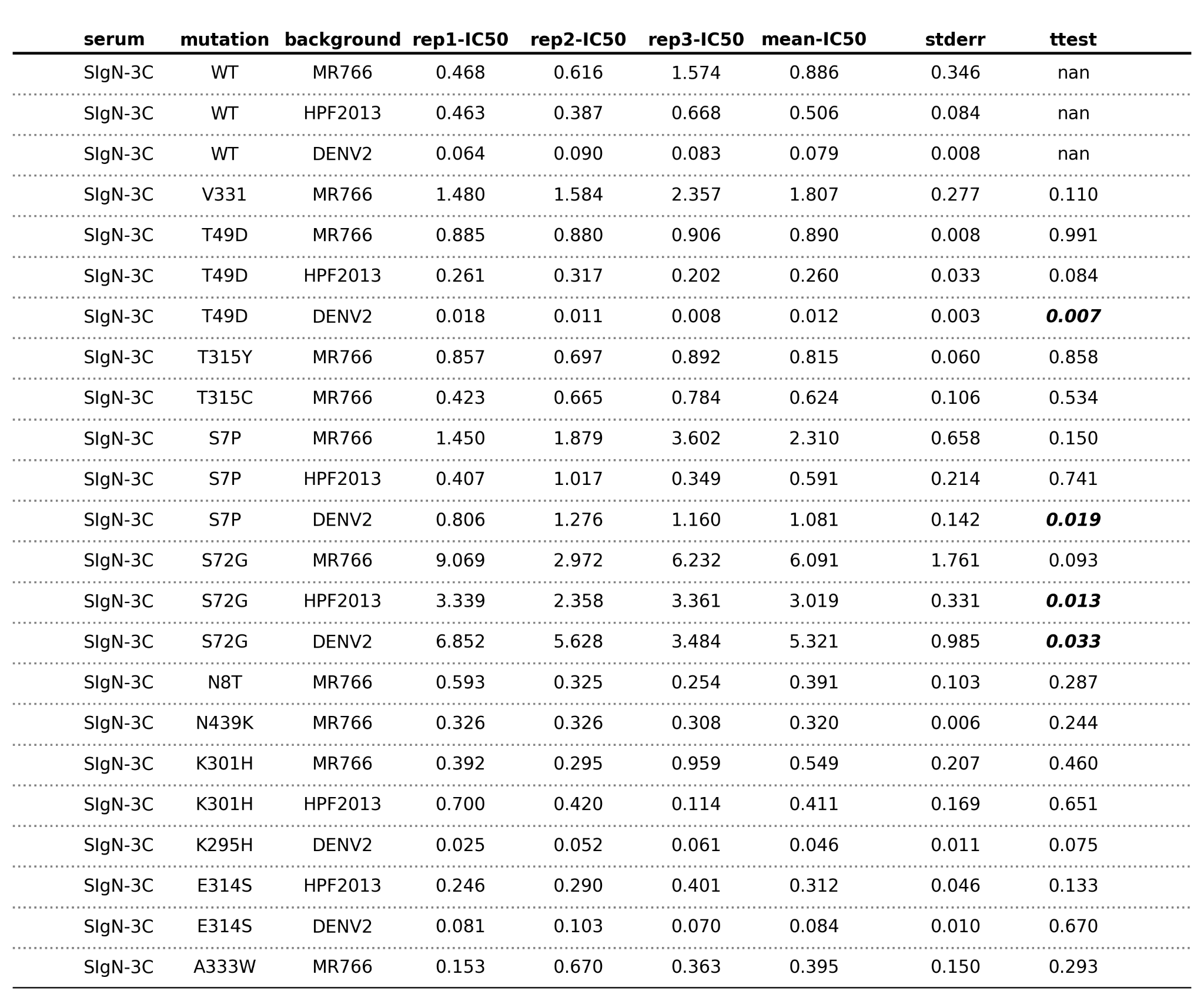


Antibodies were incubated with viruses containing the indicated mutation created in the indicated viral background. Hill curves and IC50s were inferred in technical triplicate, and the mean and standard error (stderr) were calculated. To assess for differences between wild-type (WT) and mutant virus IC50s, a Student’s T test was performed (ttest). See **Methods** for details.
